# Frequent epigenetic inactivation of secreted frizzled-related protein 2 (SFRP2) by promoter methylation in human gastric cancer

**DOI:** 10.1038/sj.bjc.6603968

**Published:** 2007-09-11

**Authors:** Y Y Cheng, J Yu, Y P Wong, E P S Man, K F To, V X Jin, J Li, Q Tao, J J Y Sung, F K L Chan, W K Leung

**Affiliations:** 1 Institute of Digestive Disease, Faculty of Medicine, The Chinese University of Hong Kong, Hong Kong, China; 2 State Key Laboratory in Oncology in South China, The Chinese University of Hong Kong, Hong Kong, China; 3 Li Ka Shing Institute of Health Sciences, The Chinese University of Hong Kong, Hong Kong, China; 4 Department of Pharmacology and the Genome Center, University of California-Davis, CA, USA; 5 Department of Clinical Oncology, The Chinese University of Hong Kong, Hong Kong, China

**Keywords:** DNA methylation, gene expression, human gastric cancer, *SFRP*s

## Abstract

The role of secreted frizzled-related protein (*SFRP*) genes in gastric cancer remains largely unknown. We determined the frequency and functional significance of *SFRP*s hypermethylation in human gastric cancer. The expression and methylation status of four *SFRP* members (*SFRP*1, 2, 4, and 5) in primary gastric cancer samples was screened. The biological effects of *SFRP* were analysed by flow cytometry, cell viability assay and *in vivo* tumour growth in nude mice. Among the four *SFRP*s, only *SFRP*2 was significantly downregulated in gastric cancer as compared to adjacent non-cancer samples (*P*<0.01). Promoter hypermethylation of *SFRP*2 was detected in 73.3% primary gastric cancer tissues, 37.5% of samples showing intestinal metaplasia and 20% adjacent normal gastric tissues. Bisulphite DNA sequencing confirmed the densely methylated *SFRP*2 promoter region. Demethylation treatment restored the expression of *SFRP*2 in gastric cancer cell lines. Forced expression of *SFRP*2 induced cell apoptosis, inhibited proliferation of gastric cancer cells and suppressed tumour growth *in vivo*. Moreover, methylated *SFRP*2 was detected in 66.7% of serum samples from cancer patients but not in normal controls. In conclusion, epigenetic inactivation of *SFRP*2 is a common and early event contributing to gastric carcinogenesis and may be a potential biomarker for gastric cancer.

Epigenetic silencing of tumour-related genes due to CpG island hypermethylation has emerged as one of the most important alternations in gastric cancer development (Leung *et al*, 2001; Yu *et al*, 2003). We have previously shown that tumour-related genes including E-cadherin, p15, and p16 were frequently methylated in gastric cancer (Leung *et al*, 2001) as well as in pre-malignant gastric lesions (Leung *et al*, 2006), suggesting dysregulation in CpG-island methylation is likely to be involved in the early gastric carcinogenesis process.

Wnt proteins are secreted signalling factors with multiple functions in development and tumourigenesis (Polakis 2007). Wnt binding to frizzled receptors leads to the cytosolic stabilisation and accumulation of *β*-catenin, which translocates to the nucleus and forms a complex with T-cell factor transcription factors, thereby regulating target gene expression. Frizzled and secreted frizzled-related proteins (*SFRP*s), a family of five secreted glycoproteins, are identified as possible negative modulators of the Wnt signal transduction pathway (Heller *et al*, 2002). The SFRPs are activated through the binding of Wnt proteins to the membrane-bound frizzled receptors, leading to the stabilisation and increase in levels of the transcription factor *β*-catenin (Ilyas 2005). Activation of *SFRP*s results in constitutive activation of Wnt signalling and is associated with inhibition of apoptosis (Melkonyan *et al*, 1997; Ko *et al*, 2002), suggesting a critical role of the *SFRP* genes in the control of Wnt signal pathway. Disruption of this pathway by downregulation of the *SFRP* genes through promoter methylation has been shown in a few human cancers including colon (Suzuki *et al*, 2004), bladder (Marsit *et al*, 2005), oesophagus (Zou *et al*, 2005), lung (Fukui *et al*, 2005), and head and neck cancers (Marsit *et al*, 2006). However, the role Wnt-antagonist genes including *SFRP*1, *SFRP*2, *SFRP*4, and *SFRP*5 in gastric cancer remains poorly defined. We have previously shown that *SFRP* was expressed in most of the adjacent non-cancer gastric samples but downregulated in 16% of gastric cancer samples (To *et al*, 2001). Moreover, Kirikoshi *et al* (2001) showed that FZD2, 5, 7, 8, 9, and 10 were upregulated in gastric cancer cell lines.

In the present study, we first determined the expression level of *SFRP*s in gastric cancer and adjacent non-cancer tissues from gastric cancer patients. Secondly, we examined the methylation status of *SFRP* genes in gastric cancer and explored the functional significance of methylation-induced silencing of *SFRP* gene expression in gastric cancer cell lines both *in vitro* and *in vivo*. We further evaluated the potential use of detecting methylated *SFRP* DNA in serum as a biomarker for gastric cancer diagnosis.

## MATERIALS AND METHODS

### Human gastric samples

A total of 35 primary gastric adenocarcinomas and their adjacent non-cancer specimens, 24 benign gastric mucosa with intestinal metaplasia, and 10 normal gastric specimens from patients with normal endoscopy were obtained from the Endoscopy Centre of the Prince of Wales Hospital. All specimens were immediately snapped frozen in liquid nitrogen and stored at −80°C until processing. Serum samples were obtained from 18 gastric cancer patients and 18 normal controls without gastric cancer. All subjects gave informed consent for obtaining the study materials. The study protocol was approved by the Clinical Research Ethics Committee of the Chinese University of Hong Kong.

### Cell lines and cell culture

A total of seven gastric cancer cell lines (Kato III, MKN-28, MKN-45, SNU-1, SNU-16, AGS, and NCI-N87) were obtained from Riken Gene Bank (Japan) and American Type Culture Collection (Manassas, VA, USA). Cells were cultured in RPMI 1640 (Invitrogen, Carlsbad, CA, USA) containing 10% fetal bovine serum at 5% CO_2_, 37°C, and 95% humidity.

### Reverse transcription, polymerase chain reaction (RT–PCR) and real-time RT–PCR

Since *SFRP*3 does not contain a CpG island of more than 200 bp, we only examined the gene expression levels of *SFRP*1, *SFRP*2, *SFRP*4, and *SFRP*5 in this study. Total RNA was extracted from 14 pairs of gastric cancer specimens and 7 gastric cancer cell lines by Trizol reagent (Gibco BRL, Life Technologies, Invitrogen) according to the manufacturer's instruction. Reverse transcription reaction was performed using 2 *μ*g of total RNA with a first strand cDNA kit (Promega, Madison, WI, USA). The mRNA expression levels of the *SFRP*s were determined by PCR (PTC-200, MJ Research, Waltham, MA, USA) and quantitative real-time PCR (LightCycler, Roche Diagnostic, Germany). Probe Design software (Roche Diagnostic) was used for designing PCR primers (Table 1A). Glyceraldehyde-3-phosohate dehydrogenase (GAPDH) was used as an internal control of RNA integrity. The ratio of *SFRP*s to GAPDH expression was determined by Relative Quantification Software (Roche Diagnostic).

### Bisulphite treatment of DNA, methylation-specific polymerase chain reaction (MSP) and quantitative methylation-specific PCR

Genomic DNA was extracted from 30 pairs of gastric cancer tissues, 24 benign gastric mucosa with intestinal metaplasia, and 10 normal gastric specimens or cell lines by a DNA mini kit (Qiagen, Valencia, CA, USA). Methylation statuses of *SFRP*1, *SFRP*2, *SFRP*4, and *SFRP*5 in gastric tissues and cell lines were determined by MSP. Briefly, 2 *μ*g of genomic DNA was bisulphite-treated with Zymo DNA Modification Kit (Zymo Research, Orange, CA, USA). Bisulphite-treated DNA was used as a template for MSP and quantitative PCR by ABI 2700 thermocycler (Applied Biosystems, CA, USA) and LightCycler (Roche Diagnostic), respectively. OLIGO 6 software (Molecular Biology Insights, CO, USA) was used for designing primers specific for methylated and unmethylated alleles (Table 1B). CpGenome™ universal methylated DNA (Chemicon International Inc., CA, USA) was used as positive control for methylation and water was used as a negative control for each amplification. To quantify the amount of DNA methylation, the methylated DNA was determined by the threshold cycle number for each sample against a standard curve generated by CpGenome universal methylated DNA (Chemicon, Temecula, CA, USA). The results were expressed as the ratio of methylated DNA to that of *β*-actin. Samples with a ratio of >0.5 were considered to have a high level of DNA methylation (Chan *et al*, 2005).

### Bisulphite sequencing

For bisulphite DNA genomic sequencing, 2 *μ*l of bisulphite-treated DNA was amplified using primers list in Table 1C. The PCR products were electrophoresed and cloned into the pCR2.1–TOPO cloning vector (Invitrogen). A total of 8–10 colonies were randomly chosen for plasmid DNA extraction with Qiaprep Spin Mini kit (Qiagen). Plasmid DNA was sequenced using the ABI PRISM BigDye Terminator Cycle Sequencing Kit in ABI 3100 sequencer (Applied Biosystems). Sequencing analysis was performed by SeqScape software (Applied Biosystems).

### Construction of expression plasmids

The full-length pcDNA3.1(+)*SFRP*2 clone was made by cloning of the full-length PCR product of *SFRP*2 with PFU DNA polymerase (Invitrogen). All the plasmid sequences and orientations were confirmed by DNA sequencing.

### Western blot

A total of 20 μg of protein of each sample was loaded in 12.5% sodium dodecyl sulfate/polyacrylamide gel electrophoresis. The proteins on the polyacrylamide gel were transferred to a member for antibody incubation. Polyclonal *SFRP*2 antibody (Santa Cruz Biotechnology, Santa Cruz, CA, USA) and the corresponding secondary antibody (Santa Cruz Biotechnology) were applied before immunoblotting. Immunoblots were developed by using the enhanced chemiluminescence detection system (Amersham, Piscataway, NJ, USA) according to the manufacturer's protocol. Human *β*-actin was used as the control of protein integrity.

### Cell proliferation assay

Transiently transfected MKN45 cells with or without *SFRP*2 expression vector were selected for proliferation assay. Cells were seeded in 96-well plates. The colorimetric MTs (Promega) assay was used to measure cell numbers at 24 and 48 h. Experiment was performed in triplicates.

### Flow cytometric (FCM) detection

Cell cycle analysis was carried out by FCM. Briefly, cells transfected with or without *SFRP*2 were removed and washed twice with PBS at 24 and 48 h. The cells were then fixed in ice-cold ethanol for 1 h. The samples were concentrated by removing the ethanol and treated with 0.01% RNase (10 mg ml^−1^, Sigma, St Louis, Mo, USA) for 10 min at 37°C. Cellular DNA was stained with 0.05% propidium iodide for 20 min at 4°C in dark. The cell cycle distribution was determined using a FACScan flow cytometer (Becton Dickinson, Mountain View, CA, USA) and 10 000 cells were analysed with MultiCycle software package (Phoenix, San Diego, CA, USA).

### Nude mice assay

Transiently transfected *SFRP*2/MKN45 cells, mock-transfected cells and parental MKN45 cells were re-suspended in fresh PBS and counted. MKN45 (1 × 10^6^ cells) with or without *SFRP*2 expression vector was injected subcutaneously into the dorsal flank of nude mice (five per group). The time interval of tumour occurrence and the dimension of the tumour were recorded every 3 days until the end of week 3. The animal study protocol was approved by the Animal Experimentation Ethics Committee of the Chinese University of Hong Kong and all procedures were performed in compliance to the guidelines of the United Kingdom Co-ordinating Committee on Cancer Research.

Two perpendicular tumour diameters, width and length, were obtained with calipers and used to calculate tumour volume with the following formula: tumour volume=length × width^2^ × 0.52 (Yagi and Bekesi, 1990). The experiment was repeated three times.

### Statistical analysis

The results were expressed as mean±s.d. or percentage where appropriate. Mann–Whitney *U*-test was used to compare the two sample groups including *in vivo* tumour growth, percentage of methylation, age, sex, and the difference in mRNA levels between cancer and non-cancer specimens. Student's *t*-test was used to compare the differences of *SFRP*2 expression on the effect of cell apoptosis and cell proliferation. *χ*^2^ test was used for comparison of methylation frequency and *Helicobacter pylori* infection. All statistical calculations were done using SPSS version 11.0 for windows (SPSS Inc., Chicago, IL, USA). A *P*-value of <0.05 was taken as statistical significance.

## RESULTS

### Gene silencing and methylation status of SFRPs in primary human gastric cancers

**Figure 1:**
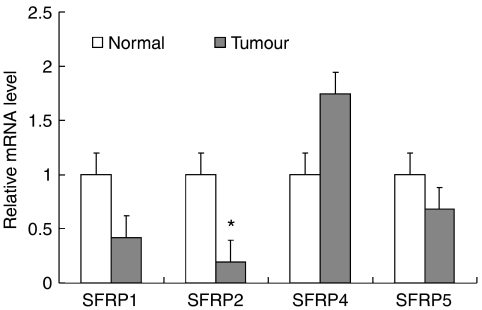
mRNA expression levels of secreted frizzled-related proteins (*SFRP*s) in primary gastric cancer (Tumour) and their adjacent non-cancer tissues (Normal) as determined by quantitative real-time PCR. The results were expressed as the ratio of copies of target gene relevant to GAPDH form three independent experiments (*n*=14). Data are expressed as mean±s.d., ^*^P<0.01.

We determined the mRNA expression of *SFRP*s in 14 pairs of primary human gastric cancers and adjacent non-cancerous tissues by quantitative RT–PCR. When compared to adjacent non-cancer tissues, *SFRP*2 was found to be significantly downregulated in primary gastric tumour specimens (Figure 1). However, this difference was not seen in *SFRP*1, *SFRP*4, and *SFRP*5.

To investigate the role of promoter methylation in silencing of *SFRP*s in human gastric cancers, the methylation status of *SFRP*s was studied in 30-paired gastric cancer specimens and their non-cancer tissue by MSP (Table 2). For *SFRP*2, 22 (73.3%) gastric cancer specimens had *SFRP*2 methylation, which was significantly higher than that of adjacent non-cancer tissue (20%, *P*<0.0001) (Table 2). There was also no significant difference in the methylation status between cancer and adjacent non-cancer areas in other *SFRP*s. Notably, *SFRP*s methylation was not seen in gastric specimens from 10 controls without gastric cancer. In addition, quantitative MSP was performed to quantify the degree of methylation in primary gastric cancer. Consistent with the semi-quantitative MSP results, the mean methylation level of *SFRP*2 was significantly higher in cancer than in adjacent non-cancer specimen (*P*<0.001). We also determined the correlation between *H. pylori* infection and *SFRP2* methylation. *H. pylori* infection was detected in 59% of gastric cancer tissues with methylation in *SFRP2* gene and 43% of gastric cancer tissues without *SFRP2* methylation (*P*=0.69). There was no association between promoter hypermethylation of *SFRP2* gene and the status of *H. pylori* infection. In addition, there was no significant association between *SFRP*2 methylation and the clinico-pathological characteristics of cancer including age, sex, tumour type, tumour differentiation, and prognosis for patients (Table 3).

### *SFRP2* expression could be restored with 5′Aza-dC treatment in gastric cancer cell lines

**Figure 2:**
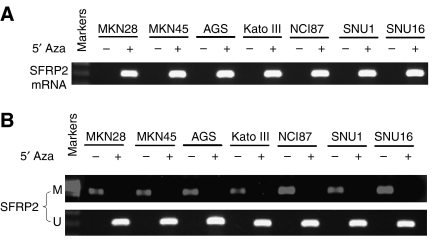
(**A**) The mRNA expression of secreted frizzled-related protein 2 (*SFRP*2) in gastric cancer cell lines (MKN28, MKN45, AGS, Kato III, NCI87, SNU1, and SNU16) treated with or without demethylation agent 5′Aza-DC as determined by RT–PCR. Pharmacologic treatment with 5′Aza-DC (5′Aza) restored the expression of *SFRP*s in tumour cell lines. (**B**) The methylation status of *SFRP*2 in gastric cancer cell lines treated with or without 5′-Aza-DC as determined by methylation specific PCR. M: methylated primers; U: unmethylated primers.

As shown in Figure 2A, *SFRP*2 was silenced in all seven human gastric cancer cell lines. We examined the role of methylation in the silencing of *SFRP*2. Using MSP, *SFRP*2 methylation was detected in all these seven cell lines with silenced expression (Figure 2B). To confirm that CpG methylation is indeed responsible for the silencing of *SFRP*2, we treated these heavily methylated and silenced cell lines with 5-Aza-DC, a methyltransferase inhibitor. *SFRP*2 expression was markedly induced after the treatment in all the cell lines (Figure 2A). These results demonstrate that CpG methylation directly contributes to the silencing of *SFRP*2 in tumour cells.

### Bisulphite sequencing of *SFRP2*

**Figure 3:**
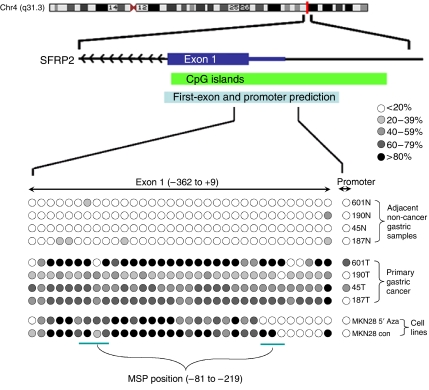
A representative picture of bisulphite sequencing in secreted frizzled-related protein 2 (*SFRP*2) gene. The CpG island, MSP region (−219 to −81) and bisulphite sequence region (−362 to +9) were shown in the upper panel. A 371-bp region with 34 CpG site was analysed. Each row of CpG sites represented an individual allele of the *SFRP*2 promoter analysed. Percentage methylation was determined as percentage of methylated cytosine from 8 to 10 randomly sequenced colonies.

To verify the MSP findings and to study the extent of promoter methylation of *SFRP*2, we performed high-resolution bisulphite genomic sequencing in the seven cell lines, four gastric cancers with low or silenced *SFRP*2 expression, and in their adjacent non-cancer tissues. The area of the CpG-rich region around the transcription initiation site of *SFRP*2 gene between the nucleotides −362 and +9 which spanned 34 CpG site was sequenced (Figure 3). There was extensive methylation in the promoter region of the MKN28 cell lines and gastric cancer tissue with low or silenced *SFRP*2 expression, whereas a very few methylated CpG sites were detected in adjacent non-cancer gastric tissues. Demethylation treatment slightly reduced the methylation density in MKN28 cells.

### *SFRP2* inhibits tumour cell proliferation and induces cell apoptosis *in vitro*

**Figure 4:**
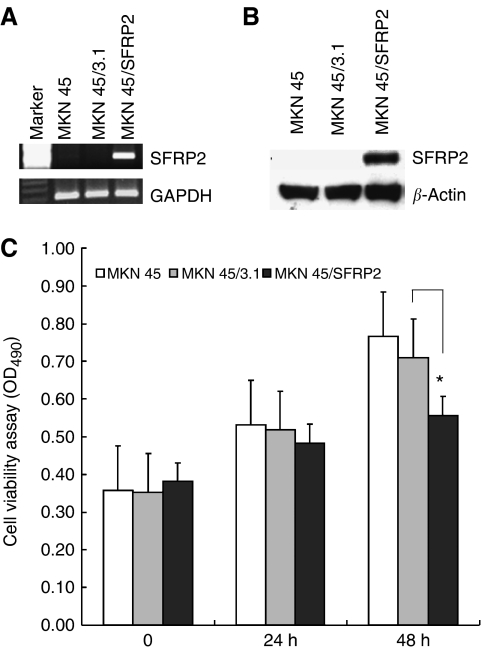
Transient transfection of secreted frizzled-related protein 2 (*SFRP*2) gene in MKN45 cell lines. (**A**) Strong *SFRP*2 mRNA expression was observed in MKN45 cells transfected with pcDNA3.1(+)*SFRP*2, but not in cells transfected with the empty vector. (**B**) Strong *SFRP*2 protein expression was observed in MKN45 cells transfected with pcDNA3.1(+)*SFRP*2, but not in cells with empty vector of in parental MKN45. (**C**) *SFRP*2 significantly suppressed cell viability at 48 h. Values are expressed as the mean±s.d. from three independent experiments (^*^P<0.001).

**Figure 5:**
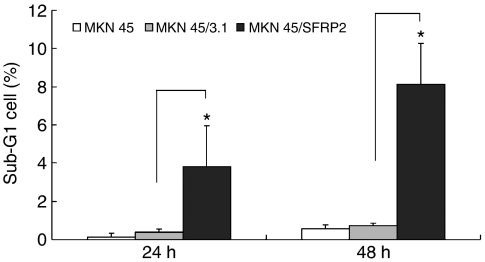
Effect of *SFRP*2 on apoptosis of human gastric cancer cells. *SFRP*2 increased the rate of apoptosis in MKN45 cells transfected with pcDNA3.1(+)*SFRP*2, as determined by the number of cells with sub-G_1_ DNA content by flow cytometry. Values are expressed as the mean±s.d. of three replicate experiments. ^*^P<0.001.

The frequent silencing of *SFRP*2 by methylation in gastric cancer but not in adjacent gastric mucosa suggests a potential tumour suppressor role of this gene. To test this speculation, we examined the effect of *SFRP*2 transfection on cell proliferation and apoptosis of gastric cancer cells. *SFRP*2 expression vector was transiently transfected into MKN45 cell line with complete methylation and silencing of *SFRP*2. Forced expression of *SFRP*2 in transfected MKN45/*SFRP*2 cells was confirmed by RT–PCR (Figure 4A) and western blot (Figure 4B). After 48 h of transfection, cell viability was reduced from 0.77 to 0.56 (*P*<0.001) (Figure 4C) as determined by cell proliferation assay. Cell apoptosis as determined by sub-G_1_ phase cells was significantly induced from 0.12 to 3.79% at 24 h (*P*<0.05) and from 0.56 to 8.1% at 48 h (*P*<0.001) (Figure 5).

### *SFRP2* inhibits gastric tumour growth *in vivo*

**Figure 6:**
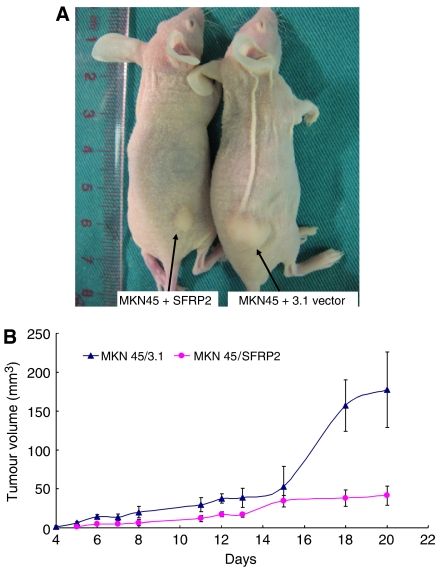
Secreted frizzled-related protein 2 (*SFRP*2) inhibits growth of tumours derived from MKN45 *in vivo*. (**A**) A representative picture of nude mice: at week 3 nude mice injected with MKN45/3.1 and MKN45/*SFRP*2. (**B**) The tumour volume of vector (pcDNA3.1) transfected MKN45 cell in nude mice (*n*=15) was indicated as mean tumour volume±s.d. (mm^3^). Tumour mean volume of MKN45/*SFRP*2 mice was significantly smaller than the MKN45/3.1 nude mice group (^*^P<0.001).

In light of the observed anti-proliferative and pro-apoptotic effects of *SFRP*2 on MKN45 *in vitro*, we tested whether *SFRP*2 would alter the growth of gastric cancer cells *in vivo* (Figure 6A). The tumour growth curve of transiently transfected MKN45/3.1-vector control and MKN45/*SFRP*2 expression vector in nude mice was shown in Figure 6B. The mean tumour volume was significantly smaller in *SFRP*2-transfected nude mice as compared to the nude mice with MKN45/3.1-vector control (*P*<0.0001).

### Methylation of *SFRP2* in pre-neoplastic gastric lesion

To determine the onset time of *SFRP*2 methylation in the gastric carcinogenesis process, we examined endoscopic gastric specimens containing foci of intestinal metaplasia, a pre-neoplastic lesion, from patients without gastric cancer. Among the 24 samples with intestinal metaplasia, 9 (37.5%) had promoter methylation in *SFRP*2 detected.

### Methylation of *SFRP2* in serum samples of gastric cancer patients

To further investigate whether *SFRP*2 methylation could be used as a biomarker for gastric cancer, serum samples of gastric cancer patients were tested for methylated *SFRP*2 DNA. A total of 12 of 18 (66.7%) serum samples from cancer patients had *SFRP*2 methylation detected whereas *SFRP*2 methylation was not detected in the serum of 18 normal subjects.

## DISCUSSION

The inappropriate activation of Wnt signalling through mutation of *β*-catenin or APC and/or downregulation of negative regulators such as *SFRPs* occurs frequently in cancers. The function of *SFRPs* as antagonist of the Wnt pathway provides a potential mechanism to suppress the abnormal activation of this pathway. Wnt signal activation by mutant *β*-catenin or APC could be suppressed partially by overexpression of *SFRP*s (Suzuki *et al*, 2002, 2004). Our previous study has shown that SFRP was downregulated in gastric cancer (To *et al*, 2001) and may carry a tumour suppressor potential. To address whether epigenetic mechanisms were responsible for SFRP downregulation, we extend the study to determine the mRNA expression level and the methylation status of *SFRP*s in gastric cancer. Among the four *SFRP*s with CpG islands in the promoter region, we found that only *SFRP*2 was silenced and methylated in gastric cancer. Promoter methylation of *SFRP*2 was specifically associated with low or absent mRNA expression in gastric cancer. The CpG island methylation status of *SFRP*2 was further confirmed by bisulphite sequencing. In addition, we confirmed that the *SFRP2* expression was restored after the demethylating agent, 5-aza-2′-deoxycytidine, treatment in gastric cancer cell lines. These data suggested that hypermethylation of the promoter region might be critical for the transcriptional silencing of *SFRP*2 in gastric cancers.

The functional role of *SFRP*2 in gastric cancer cells remains unclear and was further evaluated by examining the inhibitory effect of *SFRP*2 expression on tumour cells. Forced expression of *SFRP*2 in gastric cancer cell line MKN45 with hypermethylated *SFRP*2 inhibits cell proliferation, induces cell apoptosis, and inhibits *in vivo* tumour growth. Consistent with these findings, Suzuki *et al* (2004) demonstrated that overexpressed *SFRP*s reduced colony formation and induced apoptosis in colon cancer cells. Taken together, these data highly suggest that *SFRP*2 acts as a functional tumour suppressor gene in gastric cancer and its silencing may enhance tumour growth and expansion.

Recent epigenetic studies have suggested that silencing of the genes by DNA hypermethylation at CpG islands tended to be accumulated in the multi-step pathway of gastric carcinogenesis (Kang *et al*, 2001; To *et al*, 2002; Waki *et al*, 2002). Promoter methylation of tumour suppressor genes has been detected in early stages of gastric cancer development (Tamura 2002; Waki *et al*, 2002). In this study, methylation of *SFRP*2 was detected in intestinal metaplasia, a pre-neoplastic gastric lesion, which implicates their early involvement in the multi-step gastric carcinogenesis process. Our results showed that the frequency of hypermethylation decreased from 73.3% in gastric cancer to 37.5% in intestinal metaplasia and 20% in adjacent non-cancer tissues. It thus appears that epigenetic alteration of *SFRP*2 is present in the pre-neoplastic stage even in patients without gastric cancer, which may be an important mechanism leading to malignant transformation. Since not all intestinal metaplasia (IM) will progress to gastric cancer and promoter hypermethylation is not detected in all gastric cancers, follow-up study is required to elucidate the prognostic value of detecting promoter hypermethylation in IM. Thus, detection of hypermethylation may have the potential of identifying individuals at risk for further histological progression.

The detection of *SFRP*2 methylated DNA in serum samples of gastric cancer patients further implies their potential diagnostic value in gastric cancer. Whilst we have previously shown that methylated DNA could also be detected in cancer patients' blood (Lee *et al*, 2002; Leung *et al*, 2005), it is therefore valuable to explore the possible application of *SFRP*2 as a serum molecular biomarker for detection of human gastric cancer. We found that *SFRP*2 was detected in 67% of serum samples of cancer patients. This is higher than the previous reports in which 44–55% of gastric cancer patients had methylation detected with the use of multiple markers (Koike *et al*, 2004; Leung *et al*, 2005). Our finding suggests that *SFRP*2 is a potential source of circulating methylated DNA. Studies with larger sample size are necessary to discern the early diagnostic and prognostic significance of detecting *SFRP*2 methylation in the serum of gastric cancer patients with different subtypes.

In conclusion, we found that *SFRP*2 is hypermethylated in gastric cancer. Although widely expressed in non-cancer samples, *SFRP*2 is silenced by methylation in gastric cancer samples. We further demonstrated that *SFRP*2 could act as a functional tumour suppressor gene in gastric cancer. The high detection rate of methylated *SFRP*2 in serum indicates that *SFRP*2 methylation has its diagnostic and therapeutic values in gastric cancer.

## Figures and Tables

**Table 1:** List of primer sequences

(A) *Primer sequences for RT–PCR*	
*SFRP1*	
Sense	5′-CCAGCGAGTACGACTACGTGAGCTT-3′
Anti-sense	5′-CTCAGATTTCAACTCGTTGTCACAGG-3′
*SFRP2*	
Sense	5′-GATGATGACAACGACATAATGGAAACG-3′
Anti-sense	5′-GAGTGTGCTTGGGGAACGGGAGCT-3′
*SFRP4*	
Sense	5′-GGTACAGGAAAGGCCTCTTGATGTTG-3′
Anti-sense	5′-GGATCTTTTACTAAGCTGATCTCTCC-3′
*SFRP5*	
Sense	5′-TGCGCCCAGTGTGAGATGGAGCAC-3′
Anti-sense	5′-CCCATCCCTTAGGCCTTGTGCCAGT-3′
*GAPDH*	
Sense	5′-GGAGTCAACGGATTTGGT-3′
Anti-sense	5′-GTGATGGGATTTCCATTGAT-3′
	
(B) *Primer sequences for MSP*	
*SFRP1*	
Methylated sense	5′-TGTAGTTTTCGGAGTTAGTGTCGCGC-3′
Methylated anti-sense	5′-CCTACGATCGAAAACGACGCGAACG-3′
Unmethylated sense	5′-GTTTTGTAGTTTTTGGAGTTAGTGTTGTGT-3′
Unmethylated anti-sense	5′-CTCAACCTACAATCAAAAACAACACAAACA-3′
*SFRP2*	
Methylated sense	5′-GGGTCGGAGTTTTTCGGAGTTGCGC-3′
Methylated anti-sense	5′-CCGCTCTCTTCGCTAAATACGACTCG-3′
Unmethylated sense	5′-TTTTGGGTTGGAGTTTTTTGGAGTTGTGT-3′
Unmethylated anti-sense	5′-AACCCACTCTCTTCACTAAATACAACTCA-3′
*SFRP4*	
Methylated sense	5′-GGGTGATGTTATCGTTTTTGTATCGAC-3′
Methylated anti-sense	5′-CCTCCCCTAACGTAAACTCGAAACG-3′
Unmethylated sense	5′-GGGGGTGATGTTATTGTTTTTGTATTGAT-3′
Unmethylated anti-sense	5′-CACCTCCCCTAACATAAACTCAAAACA-3′
*SFRP5*	
Methylated sense	5′-AAGATTTGGCGTTGGGCGGGACGTTC-3′
Methylated anti-sense	5′-ACTCCAACCCGAACCTCGCCGTACG-3′
Unmethylated sense	5′-GTAAGATTTGGTGTTGGGTGGGATGTTT-3′
Unmethylated anti-sense	5′-AAAACTCCAACCCAAACCTCACCATACA-3′
*β-actin*	
Sense	5′-TGGTGATGGAGGAGGTTTAGTAAGT-3′
Anti-sense	5′-AACCAATAAAACCTACTCCTCCCTTAA-3′
	
(C) *Primer sequences for bisulphite DNA sequencing*	
*SFRP1*	
Sense	5′-TGGTTTTGTTTTTTAAGGGGTGTTGAGT-3′
Anti-sense	5′-TCCTACCRCAAACTTCCAAAAACCTCC-3′
*SFRP2*	
Sense	5′-AATTAGATTTAGAAAGTAGTGATTAGT-3′
Anti-sense	5′-AACCAAAACCCTACAACATCGTAAAC-3′
*SFRP4*	
Sense	5′-GAGGGGGTGATGTTATYGTTTTTGTAT-3′
Anti-sense	5′-CCCCAAACTCCAATCRACAACAAAAC-3′
*SFRP5*	
Sense	5′-GGGAGGTAGGGAGTTTTGGGGAGAA-3′
Anti-sense	5′-CCCAAATAAATAACAACCTACRCTAC-3′

Abbreviations: RT–PCR=reverse transcription–PCR; SFRP=secreted frizzled-related protein; GAPDH=glyceraldehyde-3-phosohate dehydrogenase; MSP=methylation-specific polymerase chain reaction.

**Table 2:** Frequency of SFRPs methylation in primary gastric samples

	**Adjacent non-tumour tissues**			**Tumour**			
	**No.**	**Methylated**	**%**	**No.**	**Methylated**	**%**	***P*-value**
SFRP1	30	25	83.3	30	27	90	>0.05
SFRP2	30	6	20	30	22	73.3	<0.0001
SFRP4	30	6	20	30	9	30	>0.05
SFRP5	30	15	50	30	9	30	>0.05

Abbreviation: SFRPs=secreted frizzled-related proteins.

**Table 3:** Patients' characteristics and methylation status

					**SFRP1**		**SFRP2**		**SFRP4**		**SFRP5**	
**Patient**	**Sex**	**Age**	**Lauren type**	***H. pylori***	**N**	**T**	**N**	**T**	**N**	**T**	**N**	**T**
1	F	78	Mix	Pos	+	+	–	+	–	–	+	–
2	M	70	—	—	+	+	–	+	+	+	+	–
3	M	54	Int	Pos	+	+	–	+	–	–	+	+
4	F	77	Diff	Pos	+	+	–	+	–	–	−	–
5	F	83	—	—	+	+	–	+	–	+	+	–
6	M	73	Diff	Neg	+	+	+	+	–	–	+	–
7	M	66	Diff	Neg	+	–	+	+	–	–	+	–
8	F	81	Diff	Neg	+	–	–	–	–	–	+	+
9	M	42	Diff	Neg	–	+	–	+	–	–	−	+
10	M	48	—	—	+	+	–	+	–	–	+	+
11	M	79	Int	Neg	+	+	+	–	+	–	+	+
12	F	72	—	—	+	+	+	+	+	+	–	+
13	F	86	Int	Pos	+	+	+	–	+	–	+	+
14	M	69	Int	Neg	+	+	–	+	–	+	–	+
15	F	69	Mix	Pos	–	+	–	+	–	–	–	–
16	M	58	—	Pos	+	–	–	+	–	–	–	–
17	M	50	Diff	neg	+	+	–	–	–	–	+	+
18	M	42	Int	Pos	+	+	–	–	–	–	+	+
19	F	80	Int	Pos	+	+	–	–	–	–	–	+
20	M	77	—	—	–	+	–	+	–	–	–	–
21	M	39	Int	Neg	+	+	–	+	–	+	+	+
22	F	32	Diff	Neg	–	+	–	–	–	–	–	+
23	M	62	Int	Pos	+	+	–	+	–	+	–	+
24	M	49	Int	Pos	+	+	–	+	–	+	–	+
25	M	57	Int	Neg	+	+	–	–	+	–	+	+
26	M	77	Int	Pos	+	+	+	+	+	–	–	+
27	M	70	Int	Neg	–	+	–	+	–	+	–	+
28	F	61	Diff	Pos	+	+	–	+	–	–	+	+
29	M	76	Int	Pos	+	+	–	+	–	+	–	+
30	F	73	Mix	Neg	+	+	–	+	–	–	–	+

Abbreviations: SFRPs=the role of secreted frizzled-related proteins; T=tumour; N=adjacent non-tumour; Int=intestinal; Diff=diffuse; Pos=positive; Neg=negative; +=methylation.
